# Koumine Attenuates Neuroglia Activation and Inflammatory Response to Neuropathic Pain

**DOI:** 10.1155/2018/9347696

**Published:** 2018-03-25

**Authors:** Gui-Lin Jin, Sai-Di He, Shao-Mei Lin, Li-Mian Hong, Wan-Qing Chen, Ying Xu, Jian Yang, Su-Ping Li, Chang-Xi Yu

**Affiliations:** ^1^Department of Pharmacology and College of Pharmacy, Fujian Medical University, Fuzhou, Fujian 350004, China; ^2^Fujian Key Laboratory of Natural Medicine Pharmacology, College of Pharmacy, Fujian Medical University, Fuzhou, Fujian, China; ^3^Department of Pharmacy, Quanzhou Medical College, Quanzhou, Fujian 362100, China

## Abstract

Despite decades of studies, the currently available drugs largely fail to control neuropathic pain. Koumine—an alkaloidal constituent derived from the medicinal plant *Gelsemium elegans* Benth.—has been shown to possess analgesic and anti-inflammatory properties; however, the underlying mechanisms remain unclear. In this study, we aimed to investigate the analgesic and anti-inflammatory effects and the possible underlying mechanisms of koumine. The analgesic and anti-inflammatory effects of koumine were explored by using chronic constriction injury of the sciatic nerve (CCI) neuropathic pain model *in vivo* and LPS-induced injury in microglia BV2 cells *in vitro*. Immunofluorescence staining and Western blot analysis were used to assess the modulator effect of koumine on microglia and astrocyte activation after CCI surgery. Enzyme-linked immunosorbent assay (ELISA) was used to evaluate the levels of proinflammatory cytokines. Western blot analysis and quantitative real-time polymerase chain reaction (qPCR) were used to examine the modulator effect of koumine on microglial M1 polarization. We found that single or repeated treatment of koumine can significantly reduce neuropathic pain after nerve injury. Moreover, koumine showed inhibitory effects on CCI-evoked microglia and astrocyte activation and reduced proinflammatory cytokine production in the spinal cord in rat CCI models. In BV2 cells, koumine significantly inhibited microglia M1 polarization. Furthermore, the analgesic effect of koumine was inhibited by a TSPO antagonist PK11195. These findings suggest that the analgesic effects of koumine on CCI-induced neuropathic pain may result from the inhibition of microglia activation and M1 polarization as well as the activation of astrocytes while sparing the anti-inflammatory responses to neuropathic pain.

## 1. Introduction

Neuropathic pain (NP) refers to pain that originates from pathological disorders of the nervous system, and both peripheral and central sensitization mechanisms can contribute to NP [1, 2]. NP due to damage to or dysfunction of the nervous system under various disease conditions affects millions of people worldwide. Hence, it is an urgent need to develop new approaches and discover new agents to treat NP.

In previous studies, effective therapy for NP focused on the primary sensory neurons and their influence on the activity of the spinal dorsal horn neurons [3]. Consistent with the progression of this neuronal mechanism, there is considerable evidence to indicate that neuroinflammation—characterized by the activation of spinal cord glial cells and the infiltration of immune cells to the nervous system—leads to the release of powerful neuromodulators such as proinflammatory cytokines and chemokines, thereof playing an important role in the induction and maintenance of NP [4]. Moreover, an inflammatory response in the spinal cord causes central sensitization of the spinal cord as manifested by long-lasting thermal hyperalgesia and mechanical allodynia [4, 5]. Thus, the disruption of glial activation and spinal cord proinflammatory cytokine action is a potentially novel treatment strategy for NP.

The 18 kDa translocator protein (TSPO)—formerly known as the peripheral-type benzodiazepine receptor (PBR)—is primarily located in the outer mitochondrial membrane. One of the most well-characterized functions of this protein is the translocation of cholesterol from the outer to inner mitochondrial membrane, which serves as the rate-limiting step in steroidogenesis [6]. However, controversy also exists regarding its role in steroidogenesis from a recent study demonstrating that siRNA-mediated knockdown of TSPO in MA-10 cells did not affect the Leydig cells' production of progesterone [7]. It is noteworthy that specific TSPO ligands such as etifoxine (Stresam) and XBD173 (AC-5216, emapunil) do stimulate the synthesis of neurosteroids and exert potent anti-inflammatory and neuroprotective effects [8, 9]. Thus, TSPO and its ligands may represent an important component of the host-defense response against disease and injury [10]. In recent years, increasing evidence has suggested that TSPO plays an important role in NP [11]. In fact, in the CNS, TSPO is reportedly expressed by activated microglia and astrocytes, which may be involved in the initiation and maintenance of NP by modulating the production of various cytokines [12, 13].

A toxic plant *Gelsemium elegans* has been used as traditional Chinese medicine for the treatment of neuralgia, sciatica, rheumatoid arthritis, and acute pain. Koumine (PubChem CID: 91895267), one of the main alkaloidal constituents of *Gelsemium elegans* Benth., has attracted an increasing amount of attention from researchers owing to its novel hexacyclic cage structure and multiple biological effects [14]. Several potential pharmaceutical roles for koumine have been identified, including anxiolytic, antitumor, antistress, antipsoriatic, and analgesic activities [14–18]. In previous studies, we found that koumine plays a significant role in the anti-inflammatory and analgesic effects in inflammatory and NP models [18–21]. Interestingly, both the anxiolytic and analgesic effects of koumine may involve the modulation of neurosteroids in the spinal cord [17–19].

In the present study, we aimed to examine the effect of koumine on spinal glial cell activation (mainly microglia and astrocyte) in a rat NP model of chronic constriction injury of the sciatic nerve (CCI). Using BV2 microglia, we assessed the effect of koumine on microglia M1 polarization. We also sought to examine whether TSPO—a mitochondrial membrane protein—is related to the analgesic effects of koumine.

## 2. Materials and Methods

### 2.1. Animals

Male Sprague-Dawley rats (Shanghai Laboratory Animal Center, Chinese Academy of Sciences), with a body weight of 200–250 g, were used in the study. In total, 6-7 animals were housed per cage and were provided ad libitum access to laboratory chow and water, except during the test periods. The rodents were maintained at a constant room temperature (25 ± 2°C), with a regular 12 : 12 h light/dark schedule, with lights on from 08:00 to 20:00 hours. The experimental protocols were approved by the ethics committee at Fujian Medical University, and the study was conducted in accordance with the guidelines published in the NIH *Guide for the Care and Use of Laboratory Animals*.

### 2.2. Surgery and Drug Administration

The rat model of CCI neuropathic pain was established in accordance with the method previously described by Bennett and Xie [22]. In brief, rats were anesthetized with chloral hydrate (400 mg/kg, i.p.). The right common sciatic nerve was dissected, exposed, and ligated at the level of the midthigh using 4 chromic gut (5-0) ties, separated by a 1 mm interval. For each ligature, a single loop was made circling the nerve and tightened to the extent that the loop was just barely snug and the ligature did not slide along the nerve. A sham operation was performed in the same manner, but without any ligation. Paw withdrawal mechanical threshold was measured using the procedures described below (in Measurement of Mechanical Hypersensitivity). Predose threshold, calculated as the CCI ipsilateral paw value/contralateral paw value, of 0.8–1.2 was considered in the study. Rats with mechanical predose threshold scores of >0.75 and/or rats that exhibited motor deficits such as hind-limb paralysis, impaired righting reflexes, and hind-limb dragging after surgery were excluded from the subsequent experiments.

Koumine (purity > 99%; HPLC) was isolated from *G. elegans* Benth. via pH-zone-refining countercurrent chromatography, which has been described in our previous study [23]. Koumine was dissolved in sterile physiological saline (0.9% NaCl) and diluted to the specified concentration before use and was then subcutaneously (s.c.) administered at a dose volume of 4 ml/kg rat body weight.

### 2.3. Intrathecal Drug Administration

Intrathecal implantation in rats has been previously described [24]. After the induction of mild anesthesia with isoflurane, the lumbar region of the rats was shaved and cleaned. Polyethylene tubing (Intramedic PE-10, Clay Adams, Parsippany, NJ) was inserted into the subarachnoid space of the lumbar enlargement. The rats that were considered neurologically healthy after intrathecal implantation for 1 day were included in the study. In contrast, the rats with locomotion deficits or without any transient motor paralysis of the hind limbs within 30 s of intrathecal injection of 2% lidocaine (20 *μ*L) were excluded from the study.

### 2.4. Measurement of Mechanical Hypersensitivity

All behavioral tests were conducted between 09:00 and 17:00 hours. Mechanical allodynia was assessed using an electronic von Frey device (series 2390; IITC Life Science Inc., Woodland Hills, CA), as described by Mitrirattanakul et al. [25] with minor modifications. Rats were acclimated for 30 min inside a Plexiglas box on a steel mesh floor, and analyses were performed using an electronic von Frey apparatus. Stimulation was applied to the center of the hind paw in an upward motion of the von Frey filament until foot withdrawal occurred, and the withdrawal threshold was automatically recorded. The test was repeated 3 times at 3–5 min intervals for each hind paw, and the average mechanical withdrawal threshold (MWT) value of each session was calculated.

### 2.5. Tissue Preparation and Immunohistochemistry

Rats were deeply anesthetized with chloral hydrate and were perfused with saline followed by paraformaldehyde through the ascending aorta (4% in 0.1 M sodium phosphate buffer; pH 7.2–7.4; 4°C). The lumbar spinal cord segments were removed and postfixed in the same fixative overnight. Tissue was then maintained in 30% sucrose in 0.1 M phosphate-buffered saline (PBS) at 4°C overnight. Dissected tissue was mounted in OCT compound and frozen at −20°C. The transverse spinal cord was cut at a thickness of 25 *μ*m in a cryostat (Microm HM 505E). For immunocytochemical analysis, the sections were washed in 0.01 M PBS 3 times (5 min each) and then blocked with 10% normal goat serum in 0.3% Triton X-100 for 1 h. After blocking, the sections were incubated overnight at 4°C in the dark with one of the following primary antibodies: anti-PBR polyclonal antibody (1 : 300; Trevigen), anti-Iba-1 polyclonal antibody (1 : 500; Abcam), and rabbit anti-GFAP polyclonal antibody (1 : 200; Abcam). The sections were then washed 3 times with PBS for 10 min each and incubated with FITC-conjugated or Cy3-conjugated antirabbit antibody (1 : 400, Jackson ImmunoResearch Laboratories Inc.) in blocking solution without Triton X-100 for 1 h in the dark. Negative staining controls were prepared by omitting either the primary antibody or secondary antibody. Fluorescence images of these sections were captured with a digital camera (Nikon 80i, Japan), and the fluorescence density was analyzed using a computer software (Image-Pro Plus 6, Media Cybernetics, USA).

### 2.6. Microglial BV2 Microglia Cultures

Microglial BV2 cells were obtained from Cell Resource Center, IBMS, CAMS/PUMC (Beijing, China) and maintained at 37°C in a humidified atmosphere with 5% CO_2_ in Dulbecco's modified Eagle's medium (DMEM, Invitrogen, CA, USA), with 10% FBS and 1% streptomycin and penicillin (Invitrogen). The culture medium was changed to a fresh medium every 2 or 3 days, and when the cells reached confluence, they were subcultured into new flasks or used immediately for the experiments. The anti-inflammatory effects of koumine appear to require extended pretreatment through the inhibition of microglial activation; therefore, in all the experiments, the cells were pretreated with the indicated concentrations of koumine or vehicle control for 12 h before the addition of LPS (1 *μ*g/ml, Sigma-Aldrich) for 24 h. The ranges of koumine concentrations were chosen based on preliminary experiments showing a good dose-effect relationship. Cell viability was determined by an MTT [3-(4,5-dimethylthiazol-2-yl)-2,5-diphenyltetrazolium bromide, Sigma-Aldrich] assay.

### 2.7. Western Blot Analysis

To examine protein expression, rats were anesthetized with sodium pentobarbital (40 mg/kg, i.p.) 8 h after the final treatment (7th dose). The L4-L5 spinal segments were quickly separated and collected in a tissue lysis buffer containing protease inhibitors, and the insoluble pellet was separated out by centrifugation (14000 ×g for 30 min, 4°C). The total protein concentration in the supernatant was measured using the Lowry method [26]. Cells were lysed with RIPA buffer (Thermo Fisher Scientific, Rockford, IL, USA), and the protein concentrations were determined using a BCA Protein Assay (Beyotime, Beijing, China). A total of 20 *μ*g of protein was loaded in each lane and was separated using a polyacrylamide gel (10%; Bio-Rad, CA, USA). After transfer, the blots were incubated overnight at 4°C with polyclonal antibody against CD68 (1 : 1000, Abcam, Cambridge, MA), polyclonal antibody against CD86 (1 : 1000, Abcam, Cambridge, MA), polyclonal antibody against TNF-*α* (1 : 1000, Cell Signaling, MA, US), polyclonal antibody against IL-1*β* (1 : 1000, Abcam, Cambridge, MA), polyclonal antibody against IL-6 (1 : 1000, Cell Signaling, MA, US), and anti-*β*-actin for loading control (1 : 2000, Cell Signaling, MA, US). These blots were further incubated with horseradish peroxidase-conjugated secondary antibody and were developed in an ECL solution (Pierce, Rockford, IL); thereafter, chemiluminescence was revealed by the Carestream Molecular Imaging system for 1–5 min. Specific bands were evaluated on the basis of the apparent molecular size. The intensity of the selected bands was analyzed using NIH ImageJ software.

### 2.8. Enzyme-Linked Immunosorbent Assay (ELISA) for TNF-*α* and IL-1*β*

The TNF-*α* and IL-1*β* concentrations in the supernatants were measured spectrophotometrically using a commercially available ELISA kit in accordance with the manufacturer's instructions (BioSite, Paris, France).

### 2.9. Quantitative Real-Time Polymerase Chain Reaction (qPCR)

Total RNA was extracted from the BV2 microglia cells using a TRIzol Reagent kit (Invitrogen, USA) in accordance with the manufacturer's recommendations. The FastStart DNA MasterPLUS SYBR Green I kit (Roche Diagnostics, Germany) was used in accordance with the manufacturer's instructions. The thermal cycling profile consisted of preincubation at 95°C for 10 min, followed by 45 cycles of 95°C for 10 s, 60°C for 30 s, and extension at 65°C for 60 s. Relative expression was calculated using the 2^−(Ct experimental sample − Ct internal control sample (GAPDH))^ method. The sequences of primers used are listed in Table 1.

### 2.10. Statistical Analysis

All data are expressed as mean ± standard error of the mean. The differences between the 2 groups at different time points were analyzed using two-way analysis of variance (ANOVA), and the differences among the different time points were analyzed using one-way ANOVA, followed by Dunnett's post hoc test. Student's *t*-test was used if only 2 groups were compared. A *P* value of <0.05 was considered statistical significance.

## 3. Results

### 3.1. Koumine Treatment Reduces Nerve Injury-Induced NP

**Figure 1:**
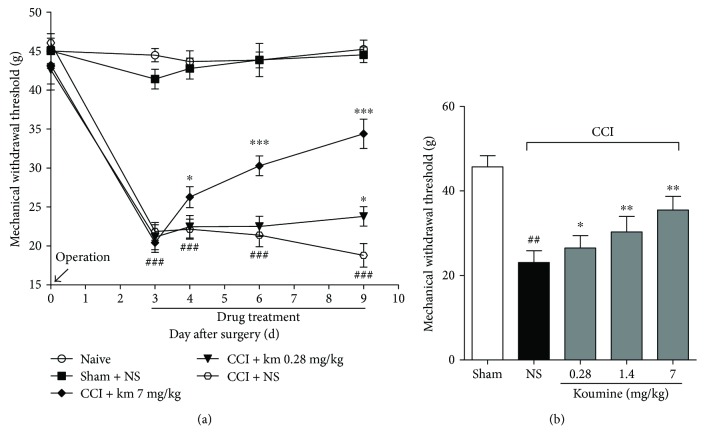
Effects of single and repeated subcutaneous administration of koumine (KM) on mechanical allodynia in rats with CCI neuropathy. (a) Koumine (0.28, 7 mg/kg) or vehicle (NS: normal saline) was subcutaneously administered once per day for 7 consecutive days from postoperative day 3. The time course of mechanical withdrawal latency with koumine showed that repeated subcutaneous injections of koumine reduced the pain behavior induced by CCI neuropathy. Data indicate the withdrawal threshold for the ipsilateral paw as mean ± SEM (*n* = 6 per group). Response of the contralateral hind paw remained unchanged throughout the procedure. ^###^*P* < 0.001 versus the sham group; ^∗^*P* < 0.05, ^∗∗∗^*P* < 0.001 versus the vehicle control group, two-way repeated-measures ANOVA followed by LSD or Dunnett's T3 test for each time point. (b) Koumine (0.28, 1.4, and 7 mg/kg) or vehicle was administered s.c. on postoperative day 9. The mechanical threshold was measured 60 min after drug administration, on the morning of postoperative day 9 for each rat. Data indicate the withdrawal threshold for the ipsilateral paw as mean ± SEM (*n* = 7–8 per group). ^##^*P* < 0.01 versus the sham group; ^∗^*P* < 0.05, ^∗∗^*P* < 0.01 versus the vehicle control group (CCI + NS), using separate one-way ANOVA followed by Bonferroni or Dunnett's T3 test for each group.

First, we assessed the effect of repeated subcutaneous administration of koumine on CCI-induced NP. Rats underwent either sham or CCI operation. Koumine (0.28, 7 mg/kg) or vehicle was administered for 7 consecutive days from postoperative day 3. Behavioral tests were performed on preoperative day 1, postoperative day 3, and 1 h after drug administration on postoperative days 5, 7, and 9. As shown in Figure 1(a), two-way repeated-measures ANOVA of the mechanical withdrawal threshold (MWT) values of the hind paw, ipsilateral to the CCI, indicated a significant therapeutic effect between subjects (*P* < 0.001) and treatment time (*P* < 0.001). Furthermore, a significant interaction was found between treatment and timing (*P* < 0.001). In these experiments, sham-operated rats displayed a small, but not significant, decrease in MWT after surgery on postoperative day 3. The hind paws of CCI-operated rats exhibited a significantly lower MWT on postoperative days 3, 4, 6, and 9 than on preoperative day 1. The administration of koumine (7 mg/kg) significantly reversed the mechanical allodynia (*P* < 0.05), as compared to the effect of the vehicle, on postoperative day 4, and the MWT increased significantly on postoperative days 6 and 9 (*P* < 0.001). Moreover, the administration of koumine (0.28 mg/kg) significantly reduced the MWT on postoperative day 9. These findings suggest that repeated subcutaneous administration of koumine can alleviate NP. Then, we examined the effects of single subcutaneous administration of koumine on mechanical allodynia in rats with CCI neuropathy. Animals with qualified predrug pain thresholds were assigned to koumine-treated groups (7 mg/kg, 1.4 mg/kg, and 0.28 mg/kg), a vehicle negative control group, or a sham control group. The MWT of each hind paw was measured 1 h after drug administration. The results showed that CCI operation significantly decreases the MWT to mechanical stimulation. In fact, single subcutaneous administration of koumine dose dependently reversed mechanical allodynia in CCI rats with an ED_50_ of 5.83 mg/kg (95% confidence limit, 3.53–13.36 mg/kg, Figure 1(b)).

### 3.2. Koumine Alters Spinal Microglia and Astrocyte Activation in the Spinal Horn of CCI Rats

**Figure 2:**
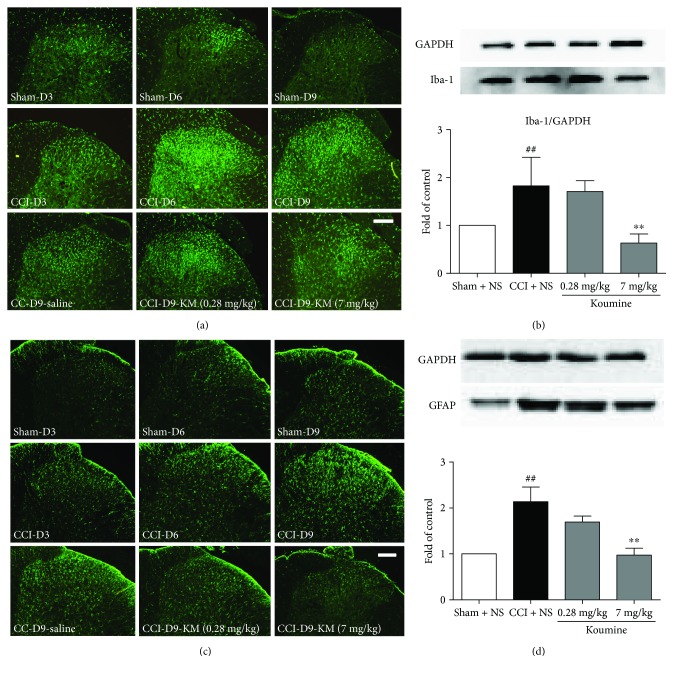
Effect of koumine treatment on microglial and astrocyte activation in the lumbar spinal cord in CCI injury rats. (a) Representative immunostaining pictures show the change in Iba-1 expression in the ipsilateral dorsal horn on days 3, 6, and 9 after CCI surgery, as well as Iba-1 expression after koumine treatment. (b) Representative bands and quantification of the Western blot analysis showed that koumine significantly suppressed the increased Iba-1 protein level on day 9. ^##^*P* < 0.01, compared with the sham + NS group; ^∗∗^*P* < 0.01, compared with the CCI + NS group, *n* = 4. (c) Representative immunostaining images show the change in GFAP expression in the ipsilateral dorsal horn on days 3, 6, and 9 after CCI surgery, as well as GFAP expression after koumine treatment. (d) Representative bands and quantification of Western blot analysis showed that koumine significantly suppressed the increased GFAP protein level on day 9. ^##^*P* < 0.01, compared with the sham + NS group; ^∗∗^*P* < 0.01, compared with the CCI + NS group, *n* = 4.

NP is associated with glial activation after nerve injury. To determine whether the administration of koumine inhibited microglia and astrocyte activation after CCI, we evaluated the protein expression of Iba-1 and GFAP at the lumbar dorsal horn via Western blot analysis and immunohistochemical staining, respectively. As shown in Figure 2(a), microglia activation, represented by the Iba-1 fluorescence density, was increased on postoperative days 3 and 6, and then slightly decreased on postoperative day 9, but remained high. CCI can also induce spinal astrocyte activation. The lumbar spinal cord sections prepared from rats on postoperative days 3, 6, and 9 exhibited an enhancement of the GFAP fluorescence density, in comparison with the spinal cord sections of naive or sham-operated rats (Figure 2(c)). In contrast to that on the ipsilateral fluorescence density, no significant extension of Iba-1 and GFAP was observed on the contralateral fluorescence density of naive or sham-operated rats (data not shown). After treatment with koumine for 7 consecutive days following CCI operation, the fluorescence density of Iba-1 and GFAP decreased on postoperative day 9. Western blot analysis confirmed that 7 mg/kg koumine significantly decreased microglial and astrocyte activation as reflected by the Iba-1 and GFAP fluorescence density in the spinal cord sections (*P* < 0.01) (Figures 2(b) and 2(d)). Microglia and astrocyte activation not only referred to specific markers (Iba-1 for microglia, GFAP for astrocyte) upregulation and astrogliosis (hypertrophy of astrocytes) but also may result in an increase of the numbers. However, there was no significant difference in the numbers of microglia between CCI rats with koumine treatment (data not shown).

### 3.3. Koumine Reduces the Production of Cytokines in the Spinal Dorsal Horn of CCI Rats

**Figure 3:**
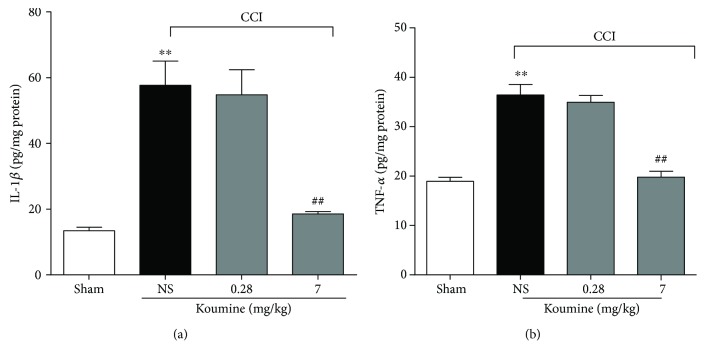
Koumine reduces the CCI-induced production of proinflammatory factors. After CCI or sham surgery, koumine (0.28, 7 mg/kg) or vehicle was administered s.c. once per day for 7 consecutive days from postoperative day 3. On postoperative day 9, the lumbar spinal cord was dissected, and the levels of IL-1*β* (a) and TNF-*α* (b) were determined by ELISA. Data are expressed as mean ± SEM (*n* = 7–12). ^∗∗^*P* < 0.01, versus the sham group; ^##^*P* < 0.01, versus the vehicle control group, separate one-way ANOVA followed by Bonferroni or Dunnett's T3 test for each group.

To further examine the spinal inflammatory response after koumine treatment, the expressions of inflammatory mediators were measured by ELISA. Koumine (0.28, 7 mg/kg, s.c.) or vehicle was administered for 7 consecutive days from postoperative day 3, and the L4-L5 spinal segments were separated after the final treatment. ELISA analyses showed that the expression of IL-1*β* and TNF-*α* in CCI-operated rats was significantly increased as compared to that in sham-operated rats (Figure 3). Treatment with koumine (7 mg/kg, s.c.) inhibited the increased production of IL-1*β* and TNF-*α* in the spinal cord of CCI-induced rats. However, there was no significant difference between sham-operated rats and CCI rats when both were treated s.c. with koumine at a dose of 0.28 mg/kg.

### 3.4. Koumine Suppresses Neuroinflammation by Inhibiting Microglia M1 Polarization in LPS-Induced BV2 Cells

**Figure 4:**
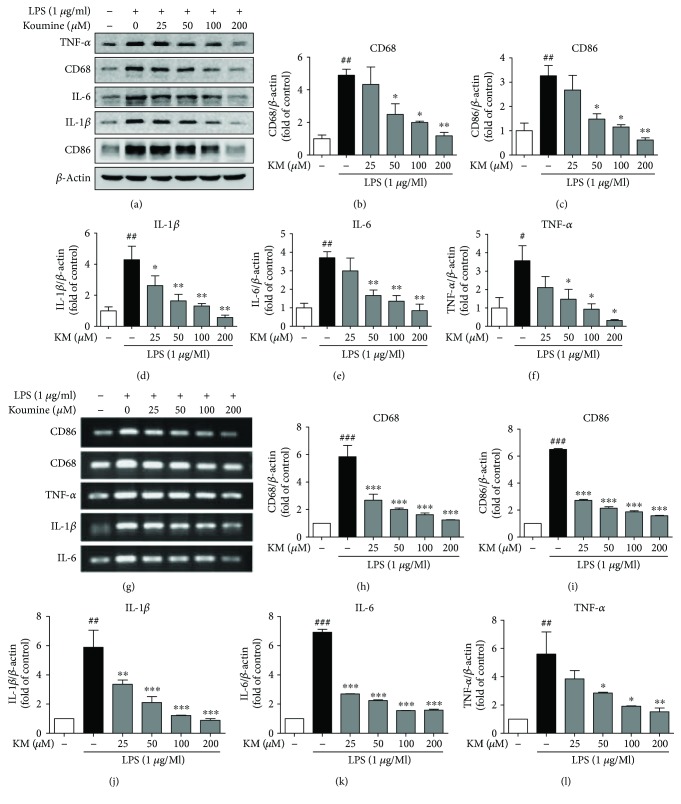
Koumine decreased the protein and mRNA levels of microglia M1 polarization factors in LPS-induced BV2 cells. BV2 cells were incubated with koumine (25, 50, 100, and 200 *μ*M) for 12 h, followed by LPS (1 *μ*g/ml) for 24 h. The protein levels of M1 markers CD86, CD68, TNF-*α*, IL-1*β*, and IL-6 were measured via Western blot analysis (a). *β*-Actin was used as a control, and the fold changes are presented (b–f). The mRNA levels of the M1 markers CD86, CD68, TNF-*α*, IL-1*β*, and IL-6 were measured using qPCR (g). GAPDH was used as the control, and the fold changes are presented (h–l). Data are presented as mean ± SEM for 3 independent experiments. ^#^*P* < 0.05, ^##^*P* < 0.01, and ^###^*P* < 0.001, compared with the control group; ^∗^*P* < 0.05, ^∗∗^*P* < 0.01, ^∗∗∗^*P* < 0.001, compared with the LPS-treated group, with one-way ANOVA followed by Bonferroni or Dunnett's T3 test for each group.

Microglia, similar to macrophages, are widely considered to adopt 2 different activation phenotypes: the proinflammatory/classically activated (M1) or anti-inflammatory/alternatively activated (M2) phenotypes. M1 cells release proinflammatory cytokines such as IL-6, IL-1*β*, and TNF-*α*, which contribute to amplifying the neuroinflammatory response. The effect of koumine on inhibiting inflammation and microglia activation led us to examine the effect of koumine on microglia M1 polarization. Therefore, we performed Western blotting and RT-PCR analysis using cell lysates and conditioned medium from koumine-treated and LPS-treated BV2 cells. We observed that the protein levels and the mRNA expression of M1 markers (CD86, CD68, IL-6, IL-1*β*, and TNF-*α*) in BV2 cells increased significantly after stimulation with LPS and reduced markedly after treatment with koumine (Figure 4).

### 3.5. Koumine Alters the Spinal TSPO Expression in the Spinal Horn of CCI Rats

**Figure 5:**
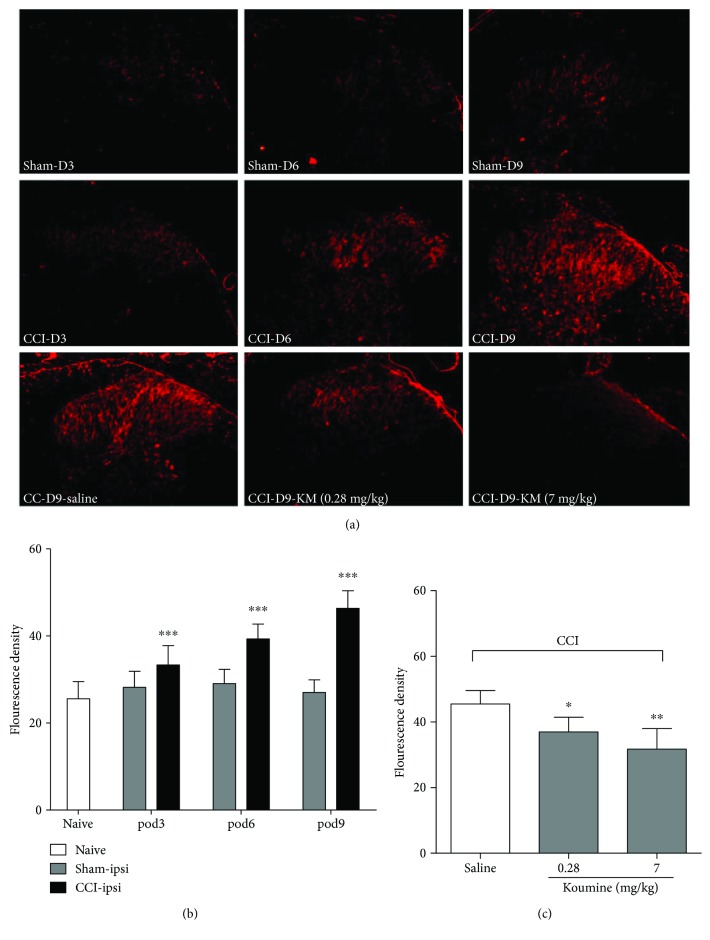
Immunohistochemical staining of TSPO expression in the spinal dorsal horn following CCI. (a) Representative experiments show the change in TSPO in the ipsilateral spinal dorsal horn from sham-operated rats and rats that received the CCI operation, as well as TSPO expression on postoperative day 9 in the ipsilateral spinal dorsal horn from CCI-operated rats after repeated treatment with koumine or saline from postoperative day 3 to day 9. *Scale bars* = 200 *μ*m. (b) The fluorescence density of the TSPO for the ipsilateral spinal dorsal horn from sham-operated rats and rats that received the CCI operation (*n* = 4). ^∗∗∗^*P* < 0.001, compared with the saline or sham group, using two-way ANOVA followed by Bonferroni or Dunnett's T3 test for each group. (c) The fluorescence density of the TSPO expression on postoperative day 9 in the ipsilateral spinal dorsal horn from CCI-operated rats after repeated treatment with koumine or saline from postoperative day 3 to day 9 (*n* = 4). ^∗^*P* < 0.05, ^∗∗^*P* < 0.01, compared with the saline or sham group, using one-way ANOVA followed by Bonferroni or Dunnett's T3 test for each group.

The translocator protein is reportedly expressed by glial cells (approximately 50% of astrocyte and 35% of microglia) and is considered to be involved in various neurological diseases, including inflammatory pain and NP [27]. To confirm the cellular effect and identify the molecular target of koumine in the CNS, we assessed the expression of TSPO in the spinal horn of CCI rats. After CCI development, the expression of TSPO changes with time (Figure 5(a)). In fact, as compared to that of the sham group, the fluorescence density of TSPO in the ipsilateral spinal cord increased significantly on postoperative day 3 (*P* < 0.001) and then significantly increased on postoperative days 6 and 9 (*P* < 0.001, Figure 5(b)). In sham-operated rats, no change was observed between the ipsilateral and contralateral spinal cords, and CCI did not affect TSPO in the contralateral spinal cord (data not shown). Interestingly, TSPO expression returned to low levels after 0.28 mg/kg (*P* < 0.05) and 7 mg/kg (*P* < 0.01) koumine administration (Figure 5(c)).

### 3.6. Blocking of the Analgesic Effect of Koumine by a TSPO Antagonist PK11195

**Figure 6:**
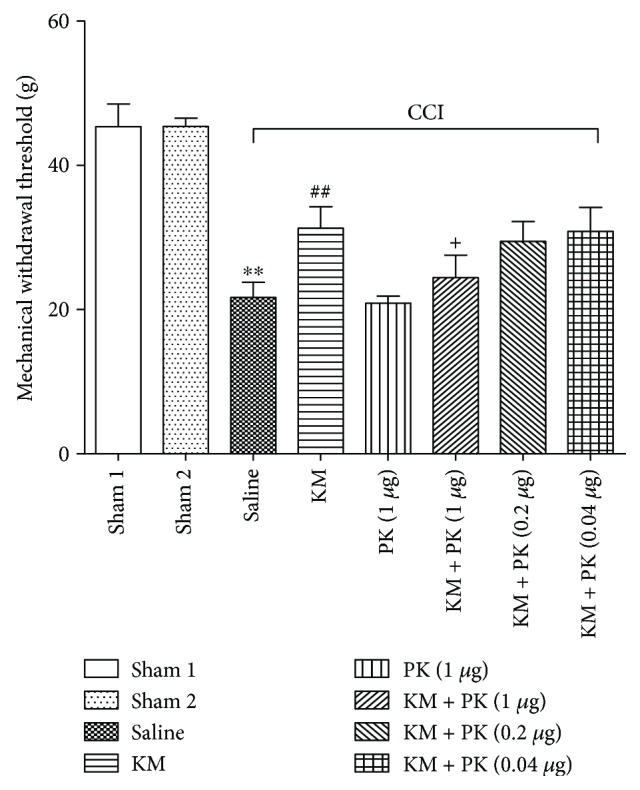
The antagonistic effect of PK11195 (PK) against the analgesic effect of koumine (KM). Koumine (7 mg/kg) was administered s.c. on postoperative day 9. PK11195 at 0.04, 0.2, or 1 *μ*g was given intrathecally immediately after koumine administration. The mechanical withdrawal threshold was assessed 60 min after intrathecal injection. Data indicate the withdrawal threshold for the ipsilateral paw as mean ± SEM (*n* = 5–7 per group). ^∗∗^*P* < 0.01, compared with the sham group. ^##^*P* < 0.01, compared with the model group. ^+^*P* < 0.05, compared with the KM group, using separate one-way ANOVA followed by Bonferroni or Dunnett's T3 test for each group.

Surgery and drug administration were performed according to the details listed in Table 2. A 1 *μ*g intrathecal injection of PK11195 significantly decreased the effect of koumine (s.c., 7 mg/kg) on the behavioral signs of NP (*P* < 0.05), indicating that koumine likely contributes to NP related to TSPO (Figure 6).

## 4. Discussion

In the present study, we observed that koumine displayed significant analgesic effects and anti-inflammatory effects and inhibited the CCI-induced activation of microglia and astrocytes in the spinal cords of rats, as well as reduced the proinflammatory cytokine levels in the spinal cord. Further examinations showed that koumine inhibited M1 microglia/macrophage polarization in BV2 cells. Moreover, the analgesic effect of koumine was found to be related to TSPO.

An increasing amount of evidence suggests that neuroinflammation in the spinal cord plays an important role in the development and maintenance of central sensitization and NP [28, 29]. Neuroinflammation contributes to chronic pain by modulating the infiltration of immune cells, activation of glial cells (mainly microglia and astrocytes), and production of inflammatory mediators in the peripheral nervous system and CNS [30, 31]. Microglia are activated in response to nerve injury and then release proinflammatory cytokines such as TNF-*α*, IL-1*β*, and IL-6, thus initiating the NP process [32, 33]. Microglia promote neuroinflammation not only by interacting with neurons but also by activating adjacent astrocytes; accordingly, the inflammatory state is prolonged, and a chronic NP condition develops [34]. The Iba-1 antibody is generally used to detect microglial activation after peripheral nerve injury via immunocytochemical staining [35]. Experiments have shown that Iba-1 expression levels are well correlated with nerve injury-induced NP and vice versa. Minocycline—an inhibitor of microglial activation—alleviates NP symptoms by suppressing microglial activation [36, 37]. Our experimental data also suggest that microglia are activated in CCI-operated rats, and that the activation is reduced following treatment with koumine, as demonstrated by Iba-1 fluorescence density and Western blot analysis of the Iba-1 protein. Once activated, microglia can exhibit M1 or M2 activation depending on the disease stage and may thus induce detrimental or beneficial effects on the nervous system [38]. Following appropriate stimulation, classically activated, proinflammatory (M1) macrophages serve as the first line of defense of the innate immune system, which often manifests within the first few hours or days [39]. These cells then produce M1-associated factors such as proinflammatory cytokines (IL-1*α*, IL-1*β*, IL-6, IL-12, IL-23, and TNF-*α*), present the antigen, and express high levels of inducible NO (iNOS) for NO production [40]. To further investigate the effect of koumine on microglia M1 polarization, LPS-induced BV2 microglia were used. Our results showed that koumine reduced the protein levels and mRNA expression of M1 markers (CD86, CD68, IL-6, IL-1*β*, and TNF-*α*) in LPS-induced BV2 microglia and downregulation of proinflammatory cytokines indicates that koumine may act at the transcriptional level to inhibit microglia M1 polarization. TSPO has been suggested to play a role in the regulation of cell nuclear gene expression. TSPO-specific ligands R-PK11195 and Ro5-4864 were reported to inhibit microglia activation and modulate the expression of proinflammatory genes and the release of cytokines [41]. Thus, we postulated that koumine inhibiting microglia M1 polarization at the transcriptional level may be related to TSPO functions. TSPO has also been suggested to play a role in apoptosis [41]. Exposure to Ro5-4864 in the presence of LPS increased the number of apoptotic microglia, suggesting that Ro-like TSPO ligands may be involved in the elimination of activated microglia via apoptosis. As a potential ligand of TSPO, koumine may get involved in the removal process of activated microglia through apoptosis, which was related with its anti-inflammatory and analgesic effects, and needs to be further researched. Except microglia, activated astrocytes are a major source of cytokines and may contribute significantly to the induction and maintenance of NP [42, 43]. Astrocytes respond to various physiological or pathological stimuli by increasing the expression of GFAP, a known marker for astrocyte activation. Through an assessment of GFAP fluorescence density and via Western blot analysis of the GFAP protein, we found that astrocytes became activated in CCI-operated rats, but astrocyte activation was reduced in the spinal cord after treatment with koumine.

Inflammation involves the body's innate immune system, including activated microglia and astrocytes, and macrophages of the CNS. On activation, these macrophages and other innate immune cells release immunoactive agents, including proinflammatory cytokines [44]. IL-1*β* is one of the most vital cytokines, and recent findings implicate IL-1*β* in painful and inflammatory processes at multiple levels, both peripherally and centrally. IL-1*β* may explain the manner in which glial cells affect neuronal activity in the CNS and promote hyperalgesia. The mediation of interactions between cells at the injury site, such as in glia and neurons, by IL-1*β* may facilitate synaptic activity and pain transmission and contribute to the development of chronic pain [45]. In addition, TNF-*α* is a key proinflammatory pain mediator as it can influence multiple mechanisms involved in pain transmission [46, 47]. TNF-*α* can also reduce the inhibitory synaptic transmission in the spinal cord, stimulate the release of additional proinflammatory cytokines, and induce the proliferation of immune and glial cells to enhance neuroinflammation and pain transmission [48]. Our results also showed that koumine reduced the CCI-induced production of cytokines. Moreover, in a recent study, koumine showed anti-inflammatory activity that is dependent on its capacity to regulate NO, IL-1*β*, IL-6, and TNF-*α* production through reduced activation of NF-*κ*B as well as p38 and ERK MAPK phosphorylation in LPS-stimulated RAW264.7 macrophages. These results imply koumine's effects on inflammatory mediator and cytokine productions [46]. Although we cannot exclude a general immunosuppressive effect of koumine by affecting M2 markers in BV2 microglia cells that may also account for its anti-inflammatory properties, it is conceivable that suppression of cytokines production by koumine, at least in part, contributes to its antinociceptive effects.

The 18 kDa translocator protein TSPO, previously known as PBR, is expressed by glial cells and neurons in the nervous system [27]. In previous studies, several TSPO agonists were found to be valuable and effective for the *in vivo* treatment of a wide range of neurological and psychiatric disorders, including pain [49, 50]. Consistent with previous studies, we found that spinal TSPO was upregulated in the dorsal horn after CCI. Moreover, the time course of TSPO signals is correlated with mechanical allodynia of behavioral tests. Although the precise underlying mechanisms remain unclear, there may be several explanations for the analgesic effects of koumine related to TSPO. First, we found that PK11195 treatment (a TSPO antagonist) is effective for inhibiting the analgesic effect of koumine in CCI-induced NP rats, and that koumine likely contributes to NP by acting on the TSPO. Second, our previous data indicated that koumine increased the levels of allopregnanolone in the spinal cord of CCI rats, thus suggesting that the anti-NP activity of koumine is mediated by the upregulation of allopregnanolone to an adequate level against NP [17]. Third, an increasing amount of evidence suggests that TSPO and its ligands may be involved in an adaptive response mechanism during neuroinflammation [51, 52]. In the present study, we found that TSPO was upregulated in the spinal dorsal horn following CCI, and that koumine treatment markedly inhibited NP. Interestingly, the upregulated TSPO was significantly diminished when the behavioral signs of NP were reversed by koumine. These results suggest that the role of TSPO upregulation might induce the recovery from the NP state. Thus, koumine can inhibit the activated microglia and astrocytes and reduce the production of inflammatory cytokines by promoting neurosteroid synthesis, thus inducing the recovery from NP and the downregulation of TSPO. It should be recognized that we did not examine whether the koumine-induced reduction of TSPO occurred at mRNA levels or through degradation of TSPO protein. Nevertheless, a study has reported that TSPO mRNA and protein levels were strongly induced in LPS-challenged BV2 microglia while the TSPO ligand XBD173 efficiently suppressed transcription of the proinflammatory marker genes.

## 5. Conclusions

In conclusion, we found that koumine reduces NP and neuroinflammation by modulating microglial activation and polarization, along with astrocyte activation. Moreover, the analgesic effects of koumine may be associated with TSPO. Thus, koumine may be a promising preventive and/or therapeutic agent for management of neuroinflammatory disorders such as NP.

## Figures and Tables

**Table 1:** Specific primers used for quantitative real-time RT-PCR (qPCR).

Gene	Forward primers	Reverse primers
GAPDH	5′-CTCGTGGAGTCTACTGGTGT-3′	5′-GTCATCATACTTGGCAGGTT-3′
CD86	5′-ACGATGGACCCCAGATGCACCA-3′	5′-GCGTCTCCACGGAAACAGCA-3′
CD68	5′-CCACAGGCAGCACAGTGGACA-3′	5′-TCCACAGCAGAAGCTTTGGCCC-3′
TNF-*α*	5′-AGCCCACGTCGTAGCAAACCAC-3′	5′-AGGTACAACCCATCGGCTGGCA-3′
IL-1*β*	5′-CCTGCAGCTGGAGAGTGTGGAT-3′	5′-TGTGCTCTGCTTGTGAGGTGCT-3′
IL-6	5′-GGAGGCTTAATTACACATGTT-3′	5′-TGATTTCAAGATGAATTGGAT-3′

**Table 2:** The experimental scheme for the antagonistic effects of PK11195 against the analgesic effects of koumine.

Group	CCI	Catheterization	s.c.	Intrathecal (PK)
Sham 1	−	−	NS	—
Sham 2	−	+	NS	Vehicle
CCI + NS	+	+	NS	Vehicle
CCI + KM	+	+	KM	Vehicle
CCI + PK	+	+	NS	PK (1 *μ*g/10 *μ*l)
CCI + KM + PK (0.04 *μ*g)	+	+	KM	PK (0.04 *μ*g/10 *μ*l)
CCI + KM + PK (0.2 *μ*g)	+	+	KM	PK (0.2 *μ*g/10 *μ*l)
CCI + KM + PK (1 *μ*g)	+	+	KM	PK (1 *μ*g/10 *μ*l)

KM: koumine; PK: PK11195; NS: normal saline.

## Abbreviations

NP: Neuropathic pain
CNS: Central nervous system
PBR: Peripheral-type benzodiazepine receptor
CCI: Chronic constriction injury of the sciatic nerve
PBS: Phosphate-buffered saline
DMEM: Dulbecco's modified Eagle's medium
ELISA: Enzyme-linked immunosorbent assay
qPCR: Quantitative real-time polymerase chain reaction
ANOVA: Analysis of variance
MWT: Mechanical withdrawal threshold
iNOS: Inducible NO.
